# Total Knee Arthroplasty Wrong Side Implant

**DOI:** 10.1055/s-0038-1673340

**Published:** 2018-09-27

**Authors:** Hassan Alanazi, Mousa Alahmari, Musaab Abdullah Hashem, Muath Alqahtani, Abdulaziz Alanazi, Bashir Alenazi

**Affiliations:** 1 Department of Orthopedic Surgery, King Faisal Specialist Hospital and Research Center, Riyadh, Saudi Arabia; 2 Senior house officer, Orthopedic Surgery, Prince Sultan Military Medical City, Riyadh, Saudi Arabia; 3 Department of Orthopedic Surgery, King Fahad Hospital, Jeddah, Saudi Arabia; 4 Department of Orthopedic Surgery, Ministry of Health, Alqurayat General Hospital, Al Qurayyat, Saudi Arabia; 5 Department of Orthopedic Surgery, Prince Sultan Military Medical City, Riyadh, Saudi Arabia

**Keywords:** arthroplasty, implant, osteoarthritis, wrong-site, knee, replacement

## Abstract

We report a unique wrong implant error during bilateral total knee replacement procedure in 72-year-old woman with bilateral knee osteoarthritis that failed conservative treatment. Patient has severe bilateral knee pain. Examination showed full range of motion. X-ray showed severe bilateral tricompartmental osteoarthritis. Patient underwent surgery in 2013; postoperative radiographs showed well-fixed femoral, tibial, and patellar components but right femoral implant was placed in the left knee. Postoperative examination showed painless full range of motion. One similar case report found describing wrong-site femoral component that ended with symptomatic. In our case, no complications were observed.

Procedures performed at the incorrect anatomical site are commonly perceived as being relatively rare. However, they can be a devastating event for patients and doctors. Evidence from the United Kingdom and North America suggests that wrong-site, wrong-procedure, and wrong-patient events occur more commonly than we think. In previous studies in North America, orthopaedic surgery has been found to be the worst-offending specialty. Wrong-site surgery is considered a devastating event to the patient, treating physician, and the institution. This event may occur in the form of wrong side, wrong level, wrong patient, wrong procedure, or wrong implant. 1 It has been termed as “never events” and refers to operating on incorrect side, incorrect level, or incorrect patient. It is estimated that these events occur 1 in every 100,000 surgical procedure. 2 Although the prevalence varies from one study to another, however, orthopaedic surgery has gained most of the attention as one of the specialties that has the highest number of wrong-site surgeries along with spine and dentistry. This occurs in orthopaedics due to the large number of cases in this specialty and working on symmetrical extremities. 3 According to a study that analyzed data from National Patient Safety Agency and National Health Services Litigation Authority on 292 cases, it was found that the most offending specialty is orthopaedics and ranked as number 1 in 2006 to 2007 in England and Wales. Wrong-site surgery is believed to be under-reported and is more common than what we think. 4

## Case Presentation

**Fig. 1:**
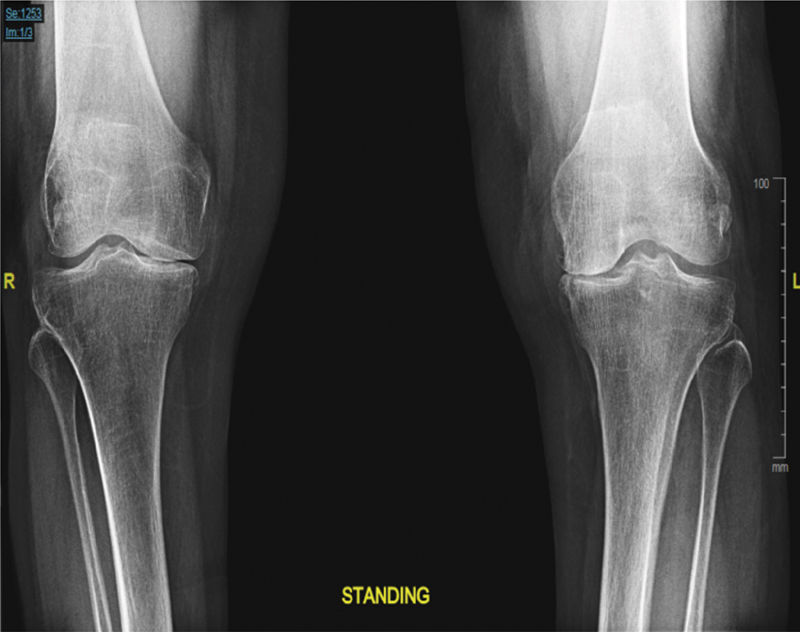
Standing anteroposterior X-ray of both knees, showing severe bilateral tricompartmental osteoarthritis.

**Fig. 2:**
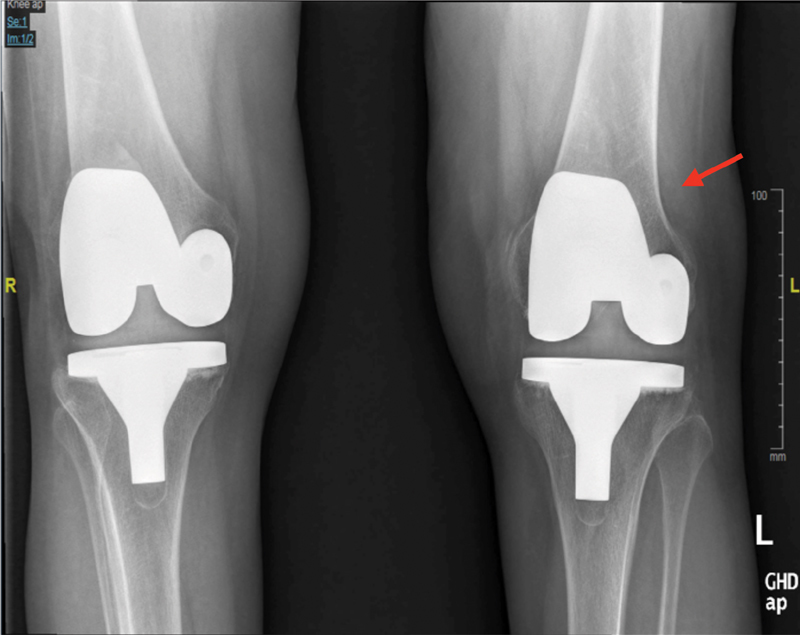
Postoperative X-ray of both knees, showing right femoral implant was placed in the left knee (arrow).

**Fig. 3:**
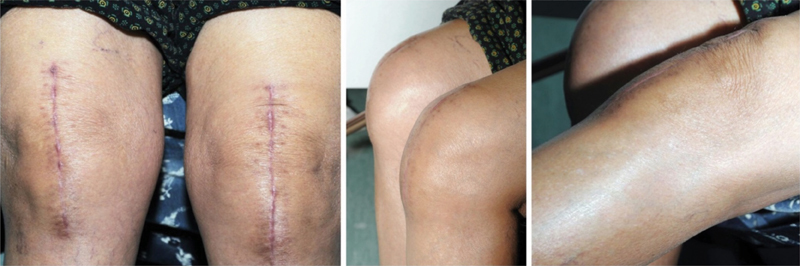
Clinical pictures during follow-up showed healed scar and full functional range of motion of both knees.

We report unique wrong implant error occurred during bilateral total knee replacement procedure in 71-year-old woman, known to have bilateral knee osteoarthritis that has failed conservative treatment. Patient reports severe pain in both knees with decreased walking distance. Examination showed full range of motion for both knees, preoperatively. X-ray showed severe bilateral tricompartmental osteoarthritis ( Fig. 1 ). Patient underwent bilateral sequential total knee replacement (PS, Sigma) in 2013. Postoperative radiographs showed well-fixed femoral, tibial, and patellar components; however, right femoral implant was placed in the left knee instead of left femoral component ( Fig. 2 ). Postoperative examination showed painless full range of motion 0 to 125 degrees. There was no patellar maltracking. No popping was heard. The patient was informed about this error. She has been following up in the clinic for 5 years. She complains of mild occasional pain but otherwise is functioning well. Knee Society score was 75. Western Ontario and McMaster Universities Osteoarthritis Index (WOMAC) was 84 ( Fig. 3 ).

## Discussion

One case report has been found in the literature describing wrong-site femoral component in total knee arthroplasty that has ended up with symptomatic patellar maltracking. 5 Sigma system is commercially available in the United Kingdom from August 1997. It has separate left and right femoral components. In our case, a right femoral component was correctly positioned in the right knee but a right femoral component was wrongly placed in the left knee ( Fig. 2 ). However, no complications have been observed in our reported case so far. During her follow-up in the clinic, WOMAC was shown to be 84 indicating very good result.

Several studies have shown that the root cause of such events is miscommunication among staff, surgical team members, ignoring members questioning the laterality of the procedure, or staff not speaking up when they notice wrong-site surgery. Other factors that may contribute are lack of time-out, lack of standardization, or lack of clear policies. It is of utmost importance to notice that proper communication inside and outside the operating room among all staff involved in the patients' care cannot be overemphasized.

The operating room is similar to an airplane cockpit, where improvements in communication through “crew resource management” have demonstrated improved safety. All members of the surgical team should feel valued and are emboldened to “speak up” and actively participate. It is the responsibility of all surgical team members to monitor and report potentially harmful situations before patient harm is caused. As with pilots and their crews' use of standardized flight procedures, the use of standardized surgical systems, including the use of checklists, is critically important to keep the patients safe. The proper implant, including the correct side, size, and implant type, compatibility, and expiration date must be confirmed before being surgically implanted to avoid medical errors and wasted implants which can exhaust the health care system budget. Implants must be opened individually during the procedure and confirmed by the entire surgical team prior to opening the package by reading the implant package label directly from a distance that allows each one of the surgical team members to properly identify the implant type, laterality, size, and expiration date. The use of large size wall mounted monitors in the operating room can help overcome the distance problem between the person presenting the package and the confirming team member. Recent novel study has presented the use of electronic labeling system has shown to improve the identification of the implants in regard to type, size, site, expiration date, and resulted in less wasted implants and can reduce the chance of wrong-site surgeries. 6

The surgeon should lead the process of procedure confirmation. If the planned surgery involves multiple surgical sites, procedures, and implants, each should be individually identified during the initial surgical “brief,” the surgical “time-out,” and the final “de-brief,” as well as confirmed individually with a “time-out” before each planned separate site, procedure, and implant. The use of a separate implant “time-out” supports focused team communication and reduces surgical errors.

## Conclusion

Wrong-site surgery is devastating event and is preventable by many measures even if it does not result in direct harm to the patient. The use of clear policies, standardization, and time-out is important. Communication must be encouraged and the slightest suspicion must be taken seriously even if it comes from a junior staff. Miscommunication is by far the commonest root cause for wrong-site surgery. New strategies should be implemented to prevent “never events.”
